# The mortality experience of disabled persons in the United States during the COVID-19 pandemic

**DOI:** 10.1093/haschl/qxad082

**Published:** 2023-12-08

**Authors:** David A Weaver

**Affiliations:** Department of Statistics, University of South Carolina, Columbia, SC 29208, United States

**Keywords:** disability, mortality, COVID-19, Social Security

## Abstract

New data from the Social Security Administration suggest there were 260 000 excess deaths in the United States among current or former disability beneficiaries during the first 22 months of the COVID-19 pandemic. These beneficiaries accounted for 26% of all excess deaths in the United States during this period. The pattern of deaths among disabled beneficiaries corresponds closely to known milestones in the pandemic's history. Disabled beneficiaries in New York, particularly those residing in institutions, had extremely elevated mortality with the onset of the pandemic in the spring of 2020. Across all regions in the United States, mortality among disability beneficiaries increased sharply with the onset of the winter of 2020–2021 and with the emergence of the Delta and Omicron variants in 2021. Elevated mortality was observed for persons with intellectual, mental, and physical impairments. Future public information campaigns about vaccines and other measures may be more successful if they include specific efforts to directly target disability beneficiaries. In addition, clinical trials and other research should consider including disabled persons as specific study groups as the severity of their underlying health impairments is likely comparable to that of persons of advanced age.

## Introduction

The mortality effects of COVID-19 on elderly individuals have been analyzed extensively,^1^ but only a small number of studies have focused on persons with disabilities. A large study in the United Kingdom found sizeable effects of COVID-19 on the mortality of persons with self-reported disabilities. That study took advantage of census data in the United Kingdom linked to official mortality records, which allowed for analysis using a very large group of individuals.^2^ Other UK-based studies found high mortality for persons with certain disabilities. Specifically, elevated mortality was observed for persons diagnosed with Down syndrome^3^ and for persons with learning disabilities.^4^

Census data linked to official mortality records are not available in the United States, but some researchers have used data from health systems to analyze outcomes for disabled persons in the United States. Analyzing patients in the Johns Hopkins Medicine System, researchers found that disability was associated with severe COVID-19, but not higher mortality. The lack of a mortality effect may have been due to small sample size.^5^ Further, some subgroups of the overall population of disabled persons have been examined in the United States. Using data from several health systems, researchers found that mortality from COVID-19 was sharply higher among persons with intellectual disabilities.^6^

The Social Security Administration (SSA) recently released person-level data that now allows for large sample analysis of mortality during the COVID-19 pandemic among persons in the United States with documented disabilities. The SSA's Disability Analysis File (DAF) contains a 10% simple random sample of all individuals who have received benefits from the Social Security Disability Insurance (SSDI) or Supplemental Security Income (SSI) disability programs in any month since 1996. The DAF includes fact and month of death through December 2021 from SSA's comprehensive system of death records.

It is possible to address an important research gap with the new SSA data—namely, to quantify the extent to which the mortality effects of the pandemic in the United States were concentrated among persons with disabilities. Specifically, the DAF can be used to estimate the number of excess deaths in the first 22 months of the pandemic (March 2020–December 2021) among disability beneficiaries. Comparing this estimate with the overall estimate of excess deaths in the United States provides policymakers with perspective and helps inform decision making in the future about appropriate allocations of public health resources to the population in the United States with disabilities.

In addition to fact and month of death, the DAF contains important information on each beneficiary, including region of residence, date of birth, primary impairment, and whether the beneficiary is institutionalized. With this information, it is possible to test an informal hypothesis that disabled persons, due to underlying health impairments, had highly elevated mortality during key moments of the pandemic. The first key moment of the pandemic in the United States was April 2020. In that month, a large number of COVID-19 deaths occurred in the country, with a strong concentration of deaths in New York City. Persons in institutions, such as nursing homes, were especially at risk during this early phase of the pandemic. The next key moment occurred in the first winter of the pandemic when vaccines were still not generally available. Deaths across all regions in the United States rose sharply. The United States next observed spikes in mortality in August and September of 2021, reflecting the spread of the Delta variant. Finally, the United States experienced a large number of deaths late in 2021 with the onset of winter and the emergence of the Omicron variant.^7^

The data in the DAF also allow for the testing of some other informal hypotheses. One key issue is whether disability in general exposed individuals to elevated mortality or whether only certain disabling impairments were associated with elevated mortality. A priori, it could be expected that disability, in general, would predict high mortality because persons with intellectual or mental impairments likely have co-occurring physical impairments. Another important hypothesis is whether or not the known age pattern of COVID-19 mortality also holds for disabled persons. A priori, it is not clear whether the pattern should hold. Children, in general, faced low mortality risks due to COVID-19, but children with disabilities have serious underlying health conditions. Further, persons with disabilities in middle age may have underlying impairments as severe as those at older ages.

The structure of this article is as follows. Using fact and month of death, the number of excess deaths among disability beneficiaries was estimated and compared with the overall number of excess deaths in the United States. Next, an analysis sample was created to test several informal hypotheses. The DAF data are described more fully in that section. Study results and limitations of the analysis are then discussed. Finally, policy and research conclusions, based on the analysis, are presented.

## Data and methods

The disability determination process used by SSA requires extensive information from the applicant, detailed medical records, and findings from disability examiners, administrative judges, medical experts, and vocational experts.^8^ Information from this process is used to create many of the variables in the DAF. The SSA also has comprehensive death information on individuals in the United States^9^ and the agency places month and year of death on the record of each individual in the DAF. The agency provides detailed documentation on the DAF, including the definitions and sources of the variables in the dataset.^10^

The SSA maintains an internal-use version of the DAF that contains the “population” of all individuals who meet the following criterion: a disability payment has been made by SSA for any month since 1996. Thus, the population in the internal-use DAF will include all current (active status) beneficiaries and all former beneficiaries for whom a payment was made in at least 1 month since 1996. The internal-use DAF is missing some former beneficiaries—namely, those whose eligibility for disability was terminated, decades ago, due to death, re-entering the workforce, or improved health. The SSA reports there are “more than 35 million” person-records in the 2021 version of the internal-use DAF.^10^

The SSA created a public-use version of the 2021 DAF, which was released in June of 2023 and which is used for this study. The public-use DAF is a 10% simple random sample of records from the internal-use DAF (the population file). The public-use file has 3.7 million person-records. To get population estimates, each record in the public-use DAF, hereafter simply referred to as the DAF, should be weighted by 10.

The DAF contains information on both of SSA's disability programs: SSI and SSDI. SSI is a means-tested program, whereas SSDI is based on payroll tax contributions. Low-income SSDI beneficiaries may also qualify for SSI. For adults, SSA uses the same definition of disability in both the SSI and SSDI programs: an inability to engage in substantial work activity due to a medically documented impairment. SSI, but generally not SSDI, also provides benefits to children (under the age of 18). The definition of disability for children is based on impaired functioning rather than an inability to work.

The SSA's disability programs have been called “strict” because they require extensive documentation and because the program does not pay any benefits if the disability is partial or temporary in nature.^11^ Research confirms both child and adult beneficiaries have serious impairments.^12,13^ The use of the DAF, therefore, allows for the study of individuals with serious and well-documented impairments.

### Excess deaths

Using fact and month of death, it is possible to determine the actual number of individuals in the DAF who died in each calendar month and year. Table 1 shows annual deaths in the DAF for the years 2017 through 2021, after weighting by 10 to reflect the DAF's 1-in-10 sampling. There is a slight positive trend in deaths before 2020 and then a sharp rise in deaths in 2020 and 2021. There are approximately 320 000 more deaths in the 2 COVID-19 years (2020 and 2021) than in the 2 years before COVID-19 (2018 and 2019). Because SSA data do not include cause of death, the increase in deaths may reflect both direct COVID-19 deaths and deaths from indirect causes, such as difficulty accessing health care^14^ during the pandemic.

**Table 1.** Deaths in the 2017–2021 period.

Year	Deaths
2017	682 900
2018	706 970
2019	712 610
2020	855 210
2021	885 810

Source: Author's tabulations from the 2021 public-use DAF. Deaths are number of deaths for each year from the DAF, weighted by 10.

Abbreviation: DAF, Disability Analysis File.

The raw numbers in Table 1 suggest that the pandemic may have brought about approximately 320 000 excess deaths among disability beneficiaries. To formally model excess deaths, however, it is necessary to specify a model of expected deaths that controls for a time trend and seasonality. A Poisson regression with a log-linear specification is used to achieve this. The model specifies that the number of deaths in a month is from a Poisson distribution whose mean (*μ*) is related to explanatory variables as follows:


(1) $$\log(\mu )=\;\alpha +\beta_{1}*\mathrm{Time}+\beta_{2}*\mathrm{January}+\cdots +\beta_{13}*\mathrm{December}$$

The variable Time is the number of months since January 2017 and the calendar-month variables are indicator (1, 0) variables. The month of January is the omitted or reference month in the estimation. The model is estimated with data from the 38 months prior to the onset of COVID-19 (January 2017 through February 2020).

Table 2 shows parameter estimates from the Poisson regression. There is a positive time trend estimated by the model, with each month after January 2017 increasing the mean number of deaths by 0.18%. There is also a strong seasonal pattern, with summer and early fall months having fewer deaths than January.

**Table 2.** Parameter estimates, Poisson regression.

	Values
Intercept	8.7572
Time	0.0018
February	−0.1143
March	−0.0562
April	−0.1164
May	−0.1240
June	−0.1844
July	−0.1573
August	−0.1599
September	−0.1872
October	−0.1304
November	−0.1133
December	−0.0663

Source: Author's tabulations from the 2021 public-use DAF. Parameter estimates are from a log-linear Poisson regression. Time reflects the number of months since January 2017 and calendar months are indicator variables (1, 0) for specific months (January is the omitted or reference month). The regression is estimated using deaths from January 2017 through February 2020. All parameter estimates are significant at the 0.001 level.

Abbreviation: DAF, Disability Analysis File.

**Figure 1.**
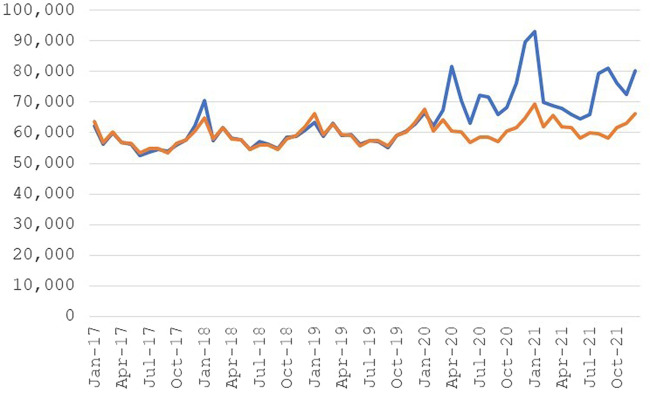
Deaths, disabled persons. Excess deaths: 261 000. Source: Author's tabulations from the 2021 public-use DAF. Actual deaths are based on total number of deaths for each month from DAF data and expected deaths are based on a Poisson regression using data from January 2017 through February 2020. Abbreviation: DAF, Disability Analysis File.

Figure 1 shows actual and expected deaths (from the model), after weighting by 10. The Poisson model fits the data well in the period before COVID-19, with expected deaths closely tracking actual deaths. With the start of the pandemic, however, the 2 series diverge sharply, with actual deaths much higher than expected. The results suggest there were about 260 000 more deaths than expected among members of the DAF universe during the first 22 months of the pandemic. This figure is 26% of the number of direct and indirect excess deaths (1 000 000) reported by the Centers for Disease Control and Prevention (CDC) for the total population in the United States during this period of the pandemic.^15^

The estimate of excess deaths among current and former disability beneficiaries (260 000) is understated because of the structure of the DAF. Former beneficiaries who never received benefits after 1996 are not included in the DAF. Such individuals would be of advanced age during the COVID-19 pandemic years and would be highly susceptible to severe outcomes from the virus.

### Analysis sample and statistical model

The baseline for the analysis that follows is December of 2018. The full analysis sample selected for this study includes all persons in the DAF who meet 2 conditions: being alive at baseline and having received a disability benefit by the time of the baseline. The second condition requires individuals, as of December 2018, to be current (active status) disability beneficiaries or former disability beneficiaries.

The full analysis sample has 2.4 million members, representing approximately 24 million persons. There are 3.7 million person-records in the DAF, but the full analysis sample excludes persons who died before the baseline and excludes persons who first became entitled to disability benefits after the baseline.

Table 3 displays descriptive statistics, by age group, for the full analysis sample. Because of the sampling structure of the DAF, persons aged 65 years or older at baseline are individuals whose initial Social Security or SSI benefit was a disability benefit that first started prior to age 65. Children, adults ages 18–64, and persons aged 65 years or older comprised 8%, 65%, and 27% of the full analysis sample, respectively.

**Table 3.** Descriptive statistics, full analysis sample by age group.

	Ages 0–17	Ages 18–64	Age 65+
Age (mean)	11.01 (0.01)	48.63 (0.01)	71.81 (0.01)
Percentages			
Died by December 2021	0.55 (0.02)	6.83 (0.02)	18.01 (0.05)
Male	64.63 (0.11)	52.33 (0.04)	47.90 (0.06)
Region			
Northeast	22.11 (0.10)	24.56 (0.03)	25.88 (0.05)
South	42.13 (0.11)	36.53 (0.04)	35.25 (0.06)
Midwest	19.14 (0.09)	21.19 (0.03)	19.03 (0.05)
West	16.62 (0.09)	17.72 (0.03)	19.85 (0.05)
Primary impairment			
Autistic	33.88 (0.11)	3.17 (0.01)	0.04 (0.00)
Intellectual	8.48 (0.06)	11.03 (0.02)	2.65 (0.02)
Mental	20.83 (0.09)	29.45 (0.04)	18.78 (0.05)
Endocrine and other systems	12.05 (0.07)	22.12 (0.03)	28.30 (0.06)
Musculoskeletal	0.73 (0.02)	23.63 (0.03)	38.18 (0.06)
Infectious and injury	1.67 (0.03)	6.69 (0.02)	7.18 (0.03)
Congenital	20.10 (0.09)	1.89 (0.01)	0.59 (0.01)
Other	2.25 (0.03)	2.01 (0.01)	4.27 (0.03)
Program			
SSI only	99.20 (0.02)	33.54 (0.04)	19.19 (0.05)
SSDI only	0.04 (0.00)	34.82 (0.04)	65.44 (0.06)
Both SSI and SSDI	0.76 (0.02)	31.63 (0.04)	15.37 (0.04)
Institutionalized	0.59 (0.02)	2.53 (0.01)	2.68 (0.02)
*n*	190 464	1 573 224	652 879

Source: Author's tabulations from the 2021 public-use DAF. Descriptive statistics, by age group, for the full analysis sample. Standard errors are in parentheses. See article text and data documentation for definitions.

Abbreviations: DAF, Disability Analysis File; SSDI, Social Security Disability Insurance; SSI, Supplemental Security Income.

Approximately 0.55% of children, 6.83% of adults ages 18–64, and 18.01% of adults 65 or older died between the baseline and December 2021. The sample size of the DAF is such that, even for rare events, the data reflect a large number of outcomes. For example, 1052 children in the analysis sample died by the end of 2021. As another example, institutionalization is relatively uncommon, but nearly 40 000 adults ages 18–64 in the analysis sample reside in institutions.

The SSA's records indicate the primary diagnosis associated with the beneficiary's disability. In the DAF released to the public, specific impairments are grouped into broader categories. Among adults ages 18–64, mental impairments are most common (29.45%), followed by musculoskeletal impairments (23.63%), endocrine and other system impairments (22.12%), intellectual disabilities (11.03%), and infectious disease and injuries (6.69%). Slightly more than half of adults ages 18–64 are male and approximately 32% have received benefits from both SSA programs (SSI and SSDI). The most common region in all age groups is the South, with a sizeable percentage of children located in this region (42.13%).

The statistical model that underlies the results in the next section of this article specifies that the logarithm of the conditional odds of death in a given month is linearly related to an intercept for the month and covariates. Specifically, the model is given as follows, where *α_t_* is the intercept for month *t*, Age is age in years at baseline, and Male is an indicator variable (1, 0) indicating gender:


(2) $$\log[P_{\mathrm{it}}/(1-P_{\mathrm{it}})]=\alpha_{t}+\beta_{1}*\mathrm{Ag}e_{i}+\beta_{2}*\mathrm{Mal}e_{i}$$

The model can be estimated after converting person-record data into person-month data.^16^ To illustrate, consider a person who dies 5 months from the baseline. The person would have 5 person-month records; a binary variable reflecting death would be equal to 0 in the first 4 records and equal to 1 in the fifth record. Note that month, year, and fact of death are available in the DAF if death occurs by December 2021; otherwise, it is censored. Thus, a person who survived through December 2021 would have 36 person-month records and the binary variable reflecting death would be equal to 0 in all 36 records. Logistic regression using the person-month data provides parameter estimates and correct standard errors for the model given in eqn (2) (see Allison^16^ for a discussion of why the person-month structure does not create dependence among the observations). For ease of exposition, the statistical model will be referred to in this article simply as logistic regression.

Odds ratios and 95% confidence intervals are reported. Odds ratios approximate relative risk ratios when the probability of an event is low.^17^ To illustrate, tabulations from the full analysis sample reveal that the probability (or risk) of death in January 2021 (conditional on surviving through December 2020) is 0.00376. The corresponding conditional risk of death in January of 2020 is 0.00264. The odds ratio is 1.43 and the relative risk ratio is 1.42. In either case it would be appropriate to say the risk of death in January 2021 was approximately 1.4 times the risk of death in January 2020.

The model is estimated for the full analysis sample and subgroups. Subgroup analysis allows for an assessment of whether key months in the pandemic's history, as discussed in the Introduction, were associated with especially high mortality among certain groups. Two demographic covariates (age in years at baseline and gender) are included in logistic regressions for the full analysis sample and for all subgroups.

## Results

### Full analysis sample

**Figure 2.**
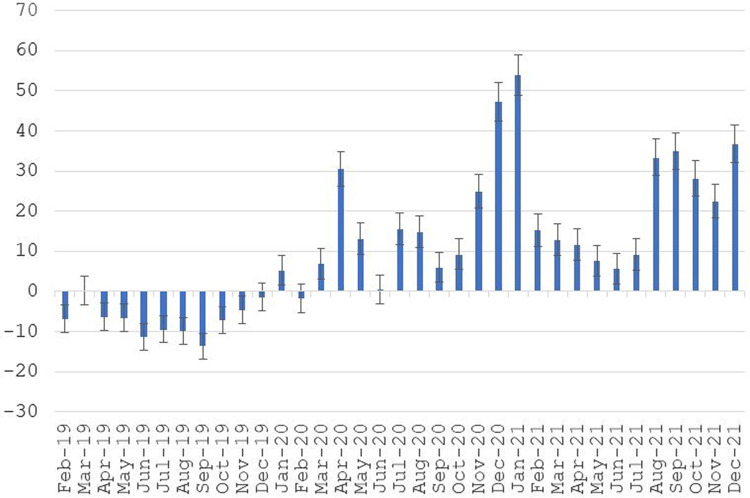
Percentage difference in odds of death relative to January 2019. Source: Author's tabulations from the 2021 public-use DAF. Odds ratios for months, measuring the odds of death for the month relative to odds of death for January 2019, were computed. Percentage differences were calculated by subtracting 1 from the odds ratio and then multiplying by 100. Based on the full analysis sample (*n* = 2 416 567). Ninety-five percent confidence intervals for percentage differences are represented by brackets. Abbreviation: DAF, Disability Analysis File.

Figure 2 displays results from estimating odds ratios, where January 2019 was the reference or omitted month. To illustrate, the odds ratio for January 2021 was estimated to be 1.54, indicating the odds of death for that month were 54% higher (shown in Figure 2) than for January 2019.

Figure 2 shows a pattern in mortality for disabled individuals that closely tracks the known history of the pandemic. There are spikes in the odds of death in April 2020, the winter months of 2020–2021, August and September of 2021, and the start of winter 2021–2022. In the 22 months in the March 2020–December 2021 period, 21 have higher odds of death. In the 13 months prior to the pandemic (February 2019–February 2020), only 1 month has (modestly) higher odds of death.

The odds ratio for males from the logistic regression is 1.39 (not shown in Figure 2), which indicates that the odds of males dying in any given month are 39% higher than the odds for females. The odds ratio for age indicates that each year of age, at baseline, increases the odds of death in any given month by approximately 6%.

Table 4 displays the results of the logistic regression using the full analysis sample in a different manner. Parameter estimates were used to develop predicted probabilities by gender for selected months and age. The probabilities were converted to monthly mortality rates (deaths per 100 000). Consistent with the odds ratio results, the mortality rate for each group is 54% higher in January 2021 than in January 2019.

**Table 4.** Monthly mortality rate (predicted).

Age (years)	Male		Female	
	January 2019	January 2021	January 2019	January 2021
35	77.8	119.7	56.1	86.4
45	134.5	206.7	97.1	149.2
55	232.3	357.0	167.7	257.8
65	401.1	615.8	289.7	445.1

Source: Author's tabulations from the 2021 public-use DAF. Parameter estimates of the logistic regression using the full analysis sample were computed and used to develop predicted probabilities of death by gender for selected months and age. The probabilities were converted to monthly mortality rates by multiplying by 100 000 (deaths per 100 000).

Abbreviation: DAF, Disability Analysis File.

### Age group

**Figure 3.**
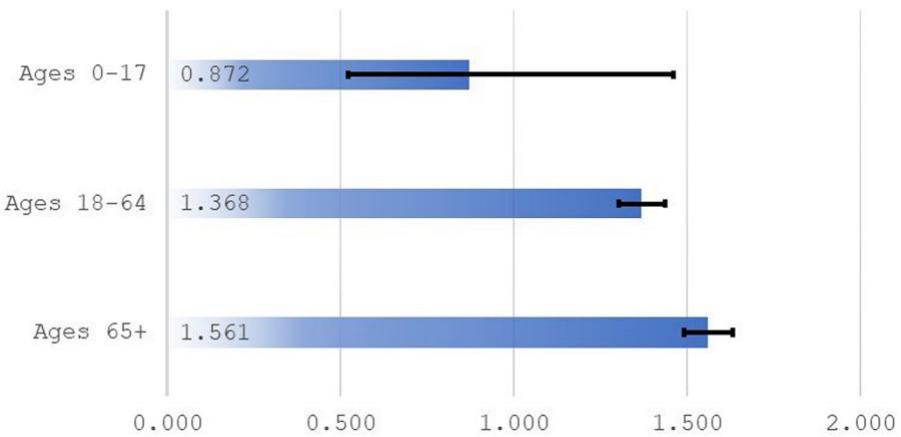
Odds ratios by age group. Source: Author's tabulations from the 2021 public-use DAF. Ratios are odds of death for January 2021 relative to odds of death for January 2020. Based on age groups from the full analysis sample. Odds ratios are from 3 separate logistic regressions (ages 0–17 [*n* = 190 464], 18–64 [*n* = 1 573 224], 65+ [*n* = 652 879]). Ninety-five percent confidence intervals for odds ratios are represented by brackets. Abbreviation: DAF, Disability Analysis File.

Logistic regressions were performed, separately, for 3 age groups, with January 2020 (a pre-pandemic month) being the omitted month. The odds ratios for January 2021 (a month during the first winter of the pandemic) are shown in Figure 3. There is not sufficient evidence to conclude there were large mortality effects from COVID-19 on children with disabilities. Disabled adults under age 65, however, were 1.37 times more likely to die in January 2021 than in January 2020. The figure for disabled persons 65 or older is higher (1.56). These results are consistent with the general age profile of COVID-19 mortality in that greater risk is associated with greater age.

The definition of disability used in this study includes both current and former disability beneficiaries (at the time of baseline). Logistic regressions (results not shown in figures) for each group separately confirmed that both groups faced elevated mortality risk during the pandemic. For current beneficiaries, the odds of an adult under the age of 65 dying in January 2021 were 1.36 times the odds from 1 year prior; the figure for former beneficiaries is 1.54. Even after exiting the rolls due to employment or somewhat improved health, former beneficiaries still likely have severe underlying health impairments and were exposed to elevated mortality risk during the pandemic.

### Region

**Figure 4.**
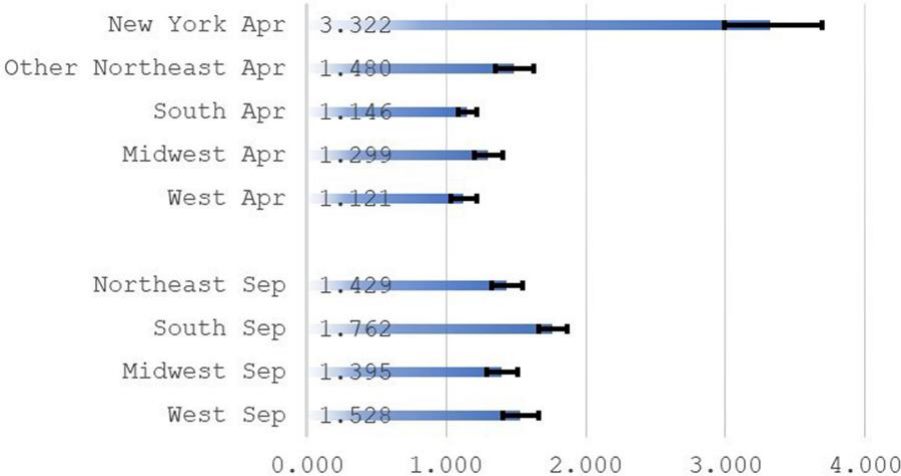
Odds ratios, selected months, by region. Source: Author's tabulations from the 2021 public-use DAF. Ratios for selected months are odds of death for April 2020 relative to odds of death for April 2019, and odds of death for September 2021 relative to odds of death for September 2019. Based on region groups from the full analysis sample. Odds ratios are from 9 separate logistic regressions (regions: New York [*n* = 243 261], other Northeast [*n* = 354 230], South [*n* = 885 089], Midwest [*n* = 493 958], West [*n* = 440 029]). Beneficiaries residing in the New York region are mainly those who reside in New York State or New Jersey. See data documentation^10^ for region definitions. Ninety-five percent confidence intervals for odds ratios are represented by brackets. Abbreviation: DAF, Disability Analysis File.

New York City was an “early epicenter” of the pandemic in the United States.^18^ Figure 4 shows the risk of death for disability beneficiaries in April 2020 relative to the risk for April 2019 by SSA administrative region. Strikingly, for disabled persons in the New York region, the risk of death in April 2020 was 3.3 times the risk of death in April 2019. The effects in other parts of the Northeast and the Midwest in the early part of the pandemic were much lower but still notable.

The Delta variant emerged in 2021 and was thought to have differential effects in the South, partly due to lower vaccination rates.^19^ For disabled persons in the South, the risk of death in September 2021, relative to the risk of death for the pre-pandemic month of September 2019, was elevated compared with other regions.

### Diagnosis

**Figure 5.**
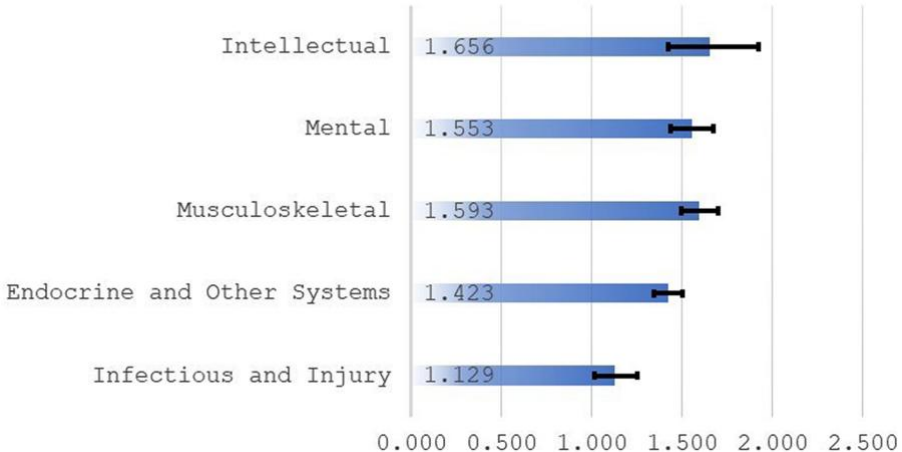
Odds ratios by diagnosis. Source: Author's tabulations from the 2021 public-use DAF. Ratios are odds of death for January 2021 relative to odds of death for January 2020. Based on selected diagnosis groups from the full analysis sample. Odds ratios are from 5 separate logistic regressions (diagnosis: Intellectual [*n* = 207 000], Mental [*n* = 625 647], Musculoskeletal [*n* = 622 418], Endocrine and Other Systems [*n* = 555 782], and Infectious and Injury [*n* = 155 321]). See data documentation^10^ for information on diagnosis groups. Individuals in these 5 diagnosis groups account for approximately 90% of all persons in the full analysis sample. Ninety-five percent confidence intervals for odds ratios are represented by brackets. Abbreviation: DAF, Disability Analysis File.

Individuals with mental impairments appear to have been strongly affected by COVID-19 during the spike in cases in the winter of 2020–2021 (Figure 5). The odds of death for persons with mental impairments were 55% higher in January 2021 than odds of death 1 year prior. Elevated mortality also occurs for persons with intellectual disabilities, which is consistent with prior research.^3,6^ The mortality effects among persons with intellectual or mental impairments were as high or higher than the effects where the primary impairment was physical. This finding illustrates that COVID-19 had strong effects on disability beneficiaries as a group, rather than beneficiaries with particular primary impairments. It is likely that disability beneficiaries are in poor health, in general, and intellectual and mental impairments co-occur with severe physical impairments.

Somewhat surprisingly, disabled persons in the infectious disease and injury group have the lowest odds ratios. Because 2 groups have been combined in the public-release data, it is not possible to determine the reasons for this. It is possible that those with an infectious disease (including those with compromised immune systems) viewed COVID-19 as particularly dangerous and took extreme precautions. Alternatively, individuals whose disabilities are due to injuries may have fewer comorbidities and faced lower risk. This important issue cannot be addressed without the provision of more granular diagnosis data from SSA.

### Vulnerable groups

**Figure 6.**
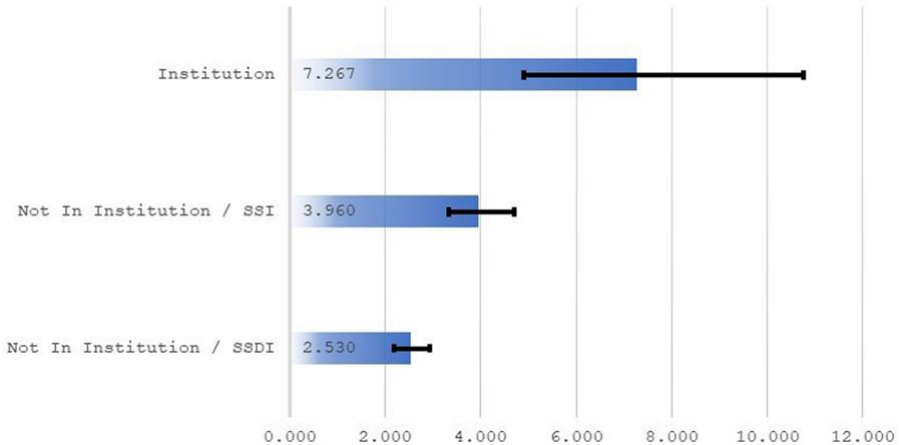
Odds ratios, New York region. Source: Author's tabulations from the 2021 public-use DAF. Ratios are odds of death for April 2020 relative to odds of death for April 2019. The sample was restricted to those from the full analysis sample that reside in the New York region. Odds ratios are from 3 separate logistic regressions (institutionalized [*n* = 8708], noninstitutionalized and SSI [*n* = 119 438], noninstitutionalized and SSDI [*n* = 115 115]). Individuals are classified as being in institutions if SSA data indicate they have a representative payee that is a state, federal, or private institution. Individuals are classified as receiving SSI if they have ever received a payment from the program; all others are classified as SSDI. Ninety-five percent confidence intervals for odds ratios are represented by brackets. Abbreviations: DAF, Disability Analysis File; SSA, Social Security Administration; SSDI, Social Security Disability Insurance; SSI, Supplemental Security Income.

During the course of the pandemic, some vulnerable groups were at extreme risk. In the early days of the pandemic in New York City, it was widely reported that persons in institutions were experiencing exceptionally high mortality.^20^ Figure 6 confirms this. The risk of death among disabled persons in institutions in the New York region in April 2020 was 7.27 times the risk of death 1 year prior.

It is important to note that disabled persons 65 or older in the analysis sample represent approximately 6.5 million individuals, which is a small fraction of the 65 or older population in the United States. Approximately 52 million persons in the United States were 65 or older in 2018.^21^ It is likely that many of the individuals who died in institutions during the early stages of the pandemic were of very advanced age. Additional data from SSA from its retirement and survivors programs would allow for a more complete analysis of the experiences of the institutionalized population during the pandemic.

Individuals with lower socioeconomic status were also widely viewed as being at elevated risk of death from COVID-19.^18^ SSI is a means-tested program and SSI recipients in the New York region faced odds of death in April 2020 that were 4 times those for April 2019. Eligibility for SSDI is not means tested; for this group, in the New York region, the odds of death in April 2020 were 2.53 times the odds in April 2019.

### Limitations

The data used in this study are from administrative records, which do not include extensive demographic information. Further, not all demographic information in SSA's record system is placed on the public-use version of the DAF. As a result, the analysis can only include limited covariates (age and gender). Further, the public-use DAF combines several detailed impairments into broad categories, which prevents the investigation of how specific impairments related to mortality during the pandemic.

The administrative data also limit how broadly disability can be defined. Some individuals with extreme disabling impairments die before receiving disability payments (left censoring). Other individuals may have disabilities that are not severe enough to qualify for SSA's programs. Overall, it is likely that the definition of disability in this study—current or prior receipt of SSDI or SSI—is a strict definition of disability. The analysis sample represents 24 million individuals, but based on survey reports in 2018, there were approximately 41 million persons in the United States who had a serious disability.^22^ Further, research indicates that disability beneficiaries in the SSDI and SSI programs have more serious impairments, on average, than beneficiaries in other disability programs in the United States, such as Workers Compensation.^12^

The strict or narrow definition of disability used in this study has implications for the findings. First, estimates from this study found that 26% of excess deaths during the first 22 months of the pandemic were among disability beneficiaries, but the percentage would be higher if all disabled persons (broadly defined) could have been studied. Second, this study found that the risk of death for disability beneficiaries was 54% higher in January 2021 than January 2019. The estimates for a broadly defined group of disabled persons would likely be lower because the underlying impairments among disability beneficiaries are likely to be severe, making them especially susceptible to serious outcomes from the virus.

A final limitation of this study is that SSA's data do not contain cause of death. As a result, it is not possible to separately identify direct and indirect effects of the pandemic.

## Discussion

The DAF offers important new data because the sample size is extremely large, the data are publicly available, and SSA's disability and death data are of high quality. Analysis of DAF data indicates that the COVID-19 pandemic had large effects on the mortality of disabled persons in the United States. These results are consistent with those of a large study of persons in the United Kingdom that used survey-reported disability status and mortality records.^2^

There are important implications for public health policy. While the elderly population was clearly identified as a specific population at risk during the pandemic, persons on SSA's disability rolls were not. Rather, vaccination recommendations and public information campaigns for the non-elderly individuals often focused on persons with specific underlying medical conditions (such as compromised immune systems) rather than severe disabilities. A potential problem with communicating with disabled persons in this manner, however, is that persons who are disabled typically have many underlying impairments and may not identify with a specific and single impairment. In this study, persons with mental impairments had highly elevated mortality, possibly due to the presence of physical comorbidities.

Future public information campaigns about vaccines and other measures may be more successful if they include specific efforts to directly target disabled persons as a group, such as those on SSDI or SSI. This population is sizeable and is at risk for serious illness and death (regardless of primary impairment). Further, this population can be reached by the federal government (SSA's administrative records include contact information for all beneficiaries).

The SSA has conducted random-assignment communication demonstrations to test whether notices to the public lead to improvements in its programs^23^ and, in principle, SSA in conjunction with federal health agencies could test whether informational notices about vaccines and other measures affect outcomes such as mortality. Communication strategies would then be evidence-based in the same manner as prevention and treatment strategies, with random-assignment studies potentially revealing causal relationships. Having effective communication strategies in place, in general, is important for mitigating the effects of future public health emergencies.

More generally, new clinical trials for vaccines or other treatments could include disabled individuals as study groups. Clinical trials often include older individuals as study groups, which results in specific recommendations. For example, research on older individuals led to the recommendations that the new respiratory syncytial virus (RSV) vaccines should be considered for persons aged 60 or older.^24^

Disability, in many cases, may be similar to advanced age in terms of underlying health. Including disabled persons as study groups in clinical trials and other research would also align with the recent, official designation of disabled persons as a “population with health disparities” by the National Institutes of Health.^25^ This designation is designed to increase research funding for studies of this population, particularly studies with a focus on how race, ethnicity, and socioeconomic status interact with underlying health problems to produce poor health outcomes.

Finally, to encourage future research, SSA could release additional data from its records. The SSA's internal-use version of the DAF includes the race of each beneficiary and less aggregated diagnosis groups, including respiratory and immune system disabilities. Inclusion of such data in future releases of the public-use file would allow additional research on how race and specific health conditions affect mortality outcomes.

Additionally, SSA could develop an Aged Analysis File. Such a file could be similar in construction to the Disability Analysis File but with beneficiaries from Social Security's very large retirement and survivor benefit programs. Such an effort would likely be inexpensive relative to other federal research initiatives (SSA uses $1.5 million from its annual research budget to produce the DAF^26^). Large-sample data on the aged would increase research on mortality generally, but would also allow for studies of small groups of particular policy interest, such as those of a very advanced age and those residing in institutional settings.

## Supplementary Material

qxad082_Supplementary_Data
